# Risk of High Dietary Calcium for Arterial Calcification in Older Adults

**DOI:** 10.3390/nu5103964

**Published:** 2013-09-30

**Authors:** John J. B. Anderson, Philip J. Klemmer

**Affiliations:** 1 Department of Nutrition, Gillings School of Global Public Health, University of North Carolina, Chapel Hill, NC 27599, USA; 2 Division of Nephrology, Department of Medicine, University of North Carolina, Chapel Hill, NC 27599, USA; E-Mail: Philip_klemmer@med.unc.edu

**Keywords:** older adults, US dietary calcium intakes, calcium supplements, calcium balance, calcium homeostasis, vitamin D, bone mineral density, skeletal health, calcium loading, arterial calcification, cardiovascular disease, chronic renal disease, osteoporosis, recommended dietary allowance for calcium

## Abstract

Concern has recently arisen about the potential adverse effects of excessive calcium intakes, *i.e.*, calcium loading from supplements, on arterial calcification and risks of cardiovascular diseases (CVD) in older adults. Published reports that high calcium intakes in free-living adults have relatively little or no beneficial impact on bone mineral density (BMD) and fracture rates suggest that current recommendations of calcium for adults may be set too high. Because even healthy kidneys have limited capability of eliminating excessive calcium in the diet, the likelihood of soft-tissue calcification may increase in older adults who take calcium supplements, particularly in those with age or disease-related reduction in renal function. The maintenance of BMD and bone health continues to be an important goal of adequate dietary calcium consumption, but eliminating potential risks of CVDs from excessive calcium intakes needs to be factored into policy recommendations for calcium by adults.

## 1. Introduction

Twenty per cent or more of adult US citizens have calcium intakes that exceed the Recommended Dietary Allowances (RDAs) [1]. Calcium supplements contribute substantially to the excessive intakes [2,3]. Spinal and hip measurements of BMI-adjusted bone mineral density (BMD) of older male and female adults of different ethnicities in the USA show little difference in BMD of the spine and hip across quintiles of calcium intakes ranging from approximately 400 to 2000 mg of calcium per day (NHANES 2005–2006) [3]. These findings suggest that high calcium intakes do not result in improved BMD, although an earlier meta-analysis demonstrated slightly improved skeletal density from higher calcium consumption [4]. In addition, several recent reports, but not all [5,6,7,8], suggest that high calcium intakes which include calcium supplements may be associated with higher rates of myocardial infarction [9,10,11,12]. Controversy exists on both issues, calcium effects on BMD and arterial calcification. Our view is that older adults may inadvertently expose themselves to higher risk of cardiovascular disease when consuming high levels of calcium. This risk may be caused by an uptake of excess calcium by ectopic bone osteoid made by phenotypic bone cells in arteries and heart valves.

Extremely high intakes of calcium, *i.e.*, >2500 mg of calcium per day, may result in hypercalcemia and the calcium-alkali syndrome [13]. Whether calcium consumption of less than 2500 mg/day contributes to arterial calcification and cardiovascular diseases (CVDs) in the general adult population is not established. Calcium loading from supplements, *i.e.*, bolus consumption of large amounts in one dose, may be more likely to contribute to arterial calcification than smaller doses from foods over a day, especially in older adults [9]. The evidence that high calcium intakes are associated with cardiovascular calcification is more compelling in those with chronic kidney disease (CKD) (see below under PTH Regulation).

This review focuses on the confluence of recent research from a number of disciplines that point to potential pathophysiological events within the arterial wall that may result from excessive calcium intakes, especially from calcium supplements. The multi-organ complexity of arterial calcification requires knowledge on many fronts to gain better understandings of the possible linkages among high calcium intake and arterial calcification and potentially adverse effects on cardiovascular health.

## 2. Setting the Stage of Arterial Calcification

Atherosclerosis is driven by the deposition of soft sub-intimal lipid-laden plaques, which tend to calcify. Beyond the normal aging process, atherosclerosis is influenced by traditional Framingham risk factors, *i.e.*, smoking, diabetes, hypertension and dyslipidemia. A growing atheroma encroaches on the arterial lumen and may lead to ischemic events including myocardial infarction and stroke if the artery becomes occluded or the plaque ruptures. The calcium component of mineralizing atherosclerotic plaques is deposited by an actively regulated process that resembles normal osteogenesis and constitutes a form of ectopic calcification. Along with the lipid component, plaque calcium contributes to luminal narrowing. Taking advantage of the fact that most atherosclerotic plaques contain calcium, rapid sequence computed tomography (CT) can be utilized to measure total atherosclerotic calcium burden by a calcium scoring technique [14]. This method has been useful in assessing the individual risk of cardiovascular events beyond that conveyed by the traditional Framingham risk factors. A number of observational studies have revealed an inverse, seemingly paradoxical, relationship between the degree of atherosclerotic burden and BMD [15,16]. Arterial calcification is considered an independent event regulated by cells, including modified smooth muscle cells, in the arterial wall [11]. Demer [17] has suggested that oxidized LDL particles may promote arterial calcification at sites where plaque formation already exists. The same oxidized LDL particles may contribute to loss of BMD.

Whether high calcium intakes, with or without calcium supplements, contribute to the calcification process in atheroma-laden arteries remains unknown. Recent cross-sectional data from a multiethnic population of older Americans ingesting a broad daily range of calcium failed to show a relationship between high calcium intakes (~1400 mg/day) and coronary artery calcification [6]. Prospective evaluation of this population is currently in progress. Two recent reports have provided data which failed to support the hypothesis that high calcium intakes increase arterial calcification in two different US populations of older adults [18,19].

Yet, retrospective studies [9,10,11,12] have provided evidence of increased incidence of acute myocardial infarction occurred among older women with high calcium intakes, especially from calcium supplements. In these reports total calcium intakes were typically in the range of 1000 to 1500 mg of calcium/day and calcium supplements contributed substantially to these totals. Other investigators did not find this association [20,21]. Neither of these studies examined calcium balance nor measured arterial calcification scores.

Clearly, resolution of these two divergent views is needed.

## 3. Adult U.S. Calcium Intakes and Recommended Dietary Allowances (RDAs)

Calcium is a threshold nutrient, which means that an adult intake greater than an estimated arbitrary upper limit, *i.e.*, the RDA, has little or no additional benefit of calcium on BMD. Table 1 lists the calcium RDAs for adults. In fact, even a calcium intake by adults well below the RDA, *i.e.*, approximating 400 to 500 mg per day may be sufficient to maintain BMD in individuals adapted to such a low intake [3]. This adaptation of an increase in enteric calcium absorption is mediated by an increase in the formation of 1,25-dihydroxycholecalciferol in the healthy functioning kidney. The Estimated Average Requirement (EAR) used by the IOM ranges from 800 to 1000 mg/day for adults [1], and based on only one report [22] the EAR may be set too high if other earlier published data are considered. For example, research by Hegsted [23], and supported in reviews [24,25,26,27], suggested that approximately 400 mg of calcium per day was sufficient on a cereal-based diet, but that probably more than 400 mg/day and not exceeding 800 mg/day was needed on a mixed Western diet containing more protein and phosphorus.

**Table 1:** Adult Recommended Dietary Allowances (RDAs) and Tolerable Upper Intake Levels (ULs) for Calcium: mg per day [1].

	RDAs		ULs	
Age Range, years	Males	Females	Males	Females
19–50	1000	1000	2500	2500
51–70	1000	1200	2000	2000
70+	1200	1200	2000	2000

Note: All EARs are set arbitrarily to be 200 mg lower than the age- and gender-specific RDAs.

A wide range of calcium intakes exists among adult males and females in the USA. A recent survey of NHANES (2004–05) intakes revealed that a majority of North Americans is meeting RDAs from a combination of food and supplemental calcium [28]. RDAs of calcium for older adults (50 years and beyond) have been set at 1000 to 1200 mg per day [1]. Another report which examined quintiles of calcium intake in older adults found that mean intakes increased from about 400 to 500 mg in the lowest quintiles by gender to greater than 2000 mg per day in the fifth quintile of two different U.S. populations [3]. Measurements of both hip and vertebral BMD, adjusted for only body mass index, were fairly consistent across all quintiles by gender and, therefore, independent of calcium intake.

Dietary supplements of calcium contribute to high calcium intakes in substantial numbers of older Americans [2,3]. In two reports, the highest consumers (quintile 5 for both males and females) ingested on average greater than 2000 mg per day [3,6]. Most of the high-consuming older adults are well educated and generally healthy, sometimes called “the worried well”, and they have sufficient incomes for obtaining bone mineral density (BMD) studies and purchasing supplements when osteopenia or osteoporosis is clinically diagnosed. Many patients do not understand that the critical time for optimal calcium intake occurs during the formative bone growth years and incorrectly assume that high calcium intakes in late life promote an increase in BMD and thereby reduce their bone fracture risk. With respect to the effect of optimal calcium intake, the window of opportunity to build strong bones closes by approximately the beginning of the third decade of life.

## 4. Physiology of Calcium Balance and Serum Calcium Homeostasis in Health and Chronic Kidney Disease

Zero calcium balance is appropriate for the adult skeleton and is achieved across a wide range of calcium intakes, even during low intakes because hormonal 1,25-dihydroxyvitamin D regulation of calcium absorption in the small intestine is enhanced. Very high calcium intakes may escape this regulatory system and, thus, contribute to an inappropriate positive calcium balance. A primary role of the kidney is to promote calcium reabsorption and retention (rather than permitting calcium excretion). Therefore, calcium balance cannot always be achieved by means of an increase of urinary excretion in those with high calcium intakes. The small intestine, however, is better adapted than the kidneys to maintain calcium balance over the entire range of customary calcium intakes [29]. Because the lowest level of enteric calcium absorption is fixed at approximately 20 percent, individuals ingesting high amounts of calcium, *i.e.*, >1500 mg/day, are at risk of developing net calcium retention [30]. Tolerable Upper Intake Levels (ULs) of calcium, as set by the IOM [1], are listed in Table 1. Assuming that calcium retention continues at levels of intake higher than those recommended, *i.e.*, >age-specific RDAs, the question arises: Where does the extra calcium go in the bodies of these adult individuals? Certainly, modest amounts of calcium are buffered temporarily in the bone fluid compartment or bone envelope (see below under Parathyroid Hormone Regulation) and some is lost in urine. Over prolonged periods of high calcium intake, however, the retained additional calcium is likely to be deposited in soft tissues and pre-existing atherosclerotic plaques (see below under Arterial Calcification) rather than in structural bone as calcium apatite. Concern has arisen that a positive calcium balance in these individuals may be expressed as soft tissue and vascular pathologic calcifications. If this hypothesis is confirmed, calcium loading may be considered as a potentially modifiable accelerant of atherosclerosis as well as the stiffening of arteries, *i.e.*, arteriosclerosis.

## 5. Parathyroid Hormone Regulation of Serum Calcium Ionic Concentration

The ionic concentration of calcium in the blood and other extracellular fluids (ECF) is tightly regulated on a minute-to-minute basis by parathyroid hormone (PTH) and it is maintained at 1.25 mM across a very wide range of dietary calcium intakes, except following supplement consumption, over a 3 to 4 h period [31]. In contrast, total body calcium balance is achieved across a wide range of dietary calcium intakes by both PTH and the slower regulatory system mediated by the action of 1,25-dihydroxyvitamin D on calcium absorption in the small intestine. Although fibroblast growth factor 23 (FGF23) is primarily involved in phosphorus balance, it also impacts on calcium balance via an inhibition of 1,25-dihydroxyvitamin D production and, indirectly, a reduction in intestinal calcium absorption. Also, FGF23 has a prompt inhibitory effect on PTH production and secretion.

Dietary calcium is too variable and intestinal absorption regulation too slow to defend this critical physiologic calcium ion set point at 1.25 mM concentration. Although the skeleton contains >99 percent of body calcium stores, only surface bone consisting of the ionizable mineral phase (but not calcium apatite) is a readily available source of calcium for these minute-to-minute regulatory requirements [32]. Instead, on the surface of all bones, beneath an envelope of epithelial cells resides a supply of readily available calcium and phosphate ions which are regulated by prompt action of PTH. When calcium intake is low, small degrees of hypocalcemia stimulate PTH secretion to cause the release of calcium into ECF, thus correcting the hypocalcemia. When calcium intakes are low, PTH also promotes calcium reabsorption by the renal tubules thus helping to maintain the physiologic set point concentration of calcium in the ECF. Conversely, during periods of high calcium intake (>1500 mg/day) and following the ingestion of a calcium supplement, PTH is suppressed as the ionic calcium ion concentration is increased [33]. The increase in serum ionic calcium permits much of the excess calcium from bolus consumption, *i.e.*, supplements, to be temporarily stored in the bone envelope, a readily available “bank” of calcium and phosphate ions [32]. Since urinary calcium excretion in normal individuals is limited to about 4 mg/kg body weight/day [34], the kidneys do not offer an adequate capability to lower the hypercalcemia during periods of high calcium intake. A potential fate of the remainder of calcium ions is uptake by atheromas that exist throughout the arterial tree, including heart valves. In fact, one report in 1997 even suggested that serum calcium concentration serves as an independent risk factor for myocardial infarcts in adult males [33].

The regulation of calcium balance of the body needs to be distinguished from the physiological regulators of the serum calcium set-point in ECF. Conceptually, calcium balance is more difficult to understand, primarily because quantitative data is derived from balance studies which are time-consuming, labor-intensive, and not readily available in the clinic. Nevertheless, numerous studies in normal subjects as well as in patients with CKD have provided useful information on this important, albeit frequently under-recognized, physiologic concept [30,35].

An external calcium balance study takes 7–10 days of inpatient study during which time the calcium content of all ingested food and supplements is measured on a daily basis. Calcium excretion is measured in feces and urine on a daily basis. An appropriate calcium balance in an adult is zero, that is, no net calcium retention occurs (as would be found in a growing child, teenager, or young adult). During years of linear skeletal growth, calcium is retained at sites of bone matrix and forms new calcium apatite. After epiphyseal closure, a typical adult ingesting 1000 mg/day of calcium will excrete approximately 800 mg/day in stool and 200 mg/day in urine, thus achieving appropriate zero calcium balance. During periods of very low dietary calcium intake, zero balance is maintained by the action of 1,25-dihydroxyvitamin D which increases intestinal calcium absorption. In addition, an increased secretion of PTH helps defend calcium balance by reducing urinary calcium excretion.

A recent balance study in normal subjects ingesting high amounts of calcium (diet plus supplements equaled 2000 mg/day) demonstrated a positive calcium balance of 450 mg/day [30]. In this study, the same calcium intake resulted in calcium retention of 750 mg/day in patients with mild CKD. In both groups, serum calcium concentrations were maintained within normal limits. Calcium retention could only be identified by means of balance studies. The sites of transfer or storage of this excess calcium, however, have not been characterized. While an increase in bone calcium apatite is unlikely, short term storage in the fluid compartment of the bone envelope is possible [32]. Over longer periods of time, however, retained calcium typically contributes to soft tissue and vascular calcification (ectopic) in older adults. These sites of calcium deposition require more quantitative evidence, particularly in populations with normal renal function, in order to complete the calcium balance equation of input equaling deposition in bone plus soft tissue calcification.

In contrast, studies in patients with CKD have generated data supporting an association between high calcium intake and cardiovascular calcification [36]. Approximately 29 million Americans have CKD [37]. CKD patients may be particularly vulnerable to vascular calcification associated with calcium loading because of both increases of calcium retention and hyperphosphatemia, two conditions that synergistically promote vascular calcification [36,38]. In this regard, it is important to emphasize that many older Americans with CKD may be at risk of experiencing accelerated vascular calcification from high calcium intakes delivered as calcium supplements in the hope of treating osteoporosis. Further studies are needed to test this hypothesis of adverse effects of high calcium intake in CKD patients.

## 6. Bone

Bone growth early in life requires protein, energy, and the micronutrients, including calcium and phosphorus, to permit successive phases of bone formation followed by bone resorption until the final bone “model” is achieved. Physical activity is well known to enhance the growth process and contribute to improved bone micro-architecture. Model formation ends when the growth plates of the long bones close. Remodeling begins typically following increases in ovarian estrogen synthesis in females and androgen production in males and this process continues to the end of life.

### 6.1. Bone Tissue and Remodeling

Bone tissue remains dynamic at all stages of the life cycle, though in late life the rate of bone turnover declines; bone mass also declines because the rate of new bone formation is less than the rate of bone resorption, a situation opposite that of the early growth years of life. Once the model is complete, normal adult bone turnover shifts first to bone resorption and then to bone formation second, processes governed by various factors made by cells in bone marrow or elsewhere, but not in bone *per se*.

Too much calcium in the diet may suppress PTH secretion and thereby reduce the pulsatile anabolic action of PTH on bone [39] and, hence, reduce the dynamics of bone turnover. Adynamic bone may occur in CKD patients exposed to high calcium intakes from calcium-based phosphate binders. The suppression of PTH by calcium loading causes a reduction in the normal anabolic effect of PTH on bone remodeling [40]. Whether this suppression occurs in subjects with high calcium intakes and normal renal function is not known.

### 6.2. Calcium Intake and Bone Status Have Little Correlation during Adulthood

Calcium supplements showed a slight improvement in BMD of adults in a meta-analysis [4], but no effect on fractures in a US Preventive Services Task Force report [41] and in one meta-analysis assessing hip fracture rate [42]. In addition, one investigator showed almost 30 years ago that Swedish women had on average the highest amount of calcium intake, one of the highest of dairy-consuming nations, and they also had one of the highest rates of hip fractures among western nations [26]. Recently, the poor inverse correlation between usual calcium intake and fractures was verified in a long-term prospective investigation of elderly Swedish women [43]. Interestingly, African Americans and most dark-skinned Africans have high bone mineral density despite low calcium intakes and low serum 25-hydroxyvitamin D concentrations.

### 6.3. Amount of Calcium Intake Limits Bone Growth, But not as Much Compared to Limited Intakes of Protein and Energy

Growth hormones that orchestrate the growth of the skeleton, muscle, and other organ systems require a supply of moderately high quality protein and sufficient energy to foster primarily the appropriate increments in height. Less than optimal intakes of either result in shorter height and often disproportionate relative growth in the torso compared to the legs, largely independent of calcium intake. If energy and protein are subsequently provided in optimal amounts during childhood, height acceleration may contribute to reasonably normal or even great height, as currently witnessed among many children in diverse nations of the world. Micronutrients, including calcium, are also required in sufficient amounts for growth, but far too often inadequate amounts are consumed.

### 6.4. Arterial Calcification in Older Adults Is Inversely Related to BMD

The co-existence of osteoporosis and variable rates of loss of BMD with increasing rates of arterial calcification occurs in older adults [15,16]. This observation signifies that the two processes are occurring simultaneously but in opposite directions. They are linked only in the sense that amounts of calcium and phosphate ions are being transferred from skeletal bone to ectopic bone formation in arterial walls made by phenotypic bone cells that have been transformed from smooth muscle cells [17,38]. Local factors seem to be acting to stimulate osteoclastic resorption in older adults, but it is not clear what proportion of the released ions is used for the supply of calcium for bone formation in arterial walls. Nevertheless, arterial walls in older adults serve as a sink for excess calcium obtained either from bone or diet or both sources, largely because of the limited capacity of the kidneys to excrete extra calcium [34].

## 7. Conclusions

Calcium intakes that consistently exceed the RDA of older adults, especially when a substantial amount of the calcium is derived from bolus intakes of calcium supplements, may accelerate arterial calcification and raise the risk of cardiovascular events. This risk is much greater in patients with CKD because of their likelihood of calcifying arteries even in the absence of calcium loading. Whether arterial calcification occurs in older adults with truly normal renal function who consume high amounts of calcium in their diets, including supplements, has not yet been determined. Further prospective investigations, especially randomized control trials, on the calcium-CVD relationship, especially in those with high calcium intakes that approach or exceed the ULs, are clearly needed. High calcium intakes from a supplement bolus have now been generally established to have little or no clinical benefit on BMD or bone-fracture rate of non-institutionalized and non-homebound older adults. Health professionals and policy makers need to re-consider their advice regarding healthy patterns of daily calcium consumption in order to maintain skeletal benefits while reducing or even eliminating potential exposure to adverse cardiovascular effects from excessive calcium intakes, and they need to continue monitoring calcium recommendations, especially when calcium is consumed from non-food sources.

## Abbreviations

BMD: bone mineral density
BMI: body mass index
CAC: coronary artery calcification
CKD: chronic kidney disease
CT: computerized tomography
CVD: cardiovascular disease
ECF: extracellular fluid
IOM: Institute of Medicine
NHANES: National Health and Nutrition Examination Survey
PTH: parathyroid hormone
FGF23: fibroblast growth factor 23
RDA: recommended dietary allowance
